# Modeling Impact of Temperature and Human Movement on the Persistence of Dengue Disease

**DOI:** 10.1155/2017/1747134

**Published:** 2017-09-19

**Authors:** Ganga Ram Phaijoo, Dil Bahadur Gurung

**Affiliations:** Department of Natural Sciences (Mathematics), School of Science, Kathmandu University, Dhulikhel, Kavre, Nepal

## Abstract

Dengue is a vector-borne infectious disease endemic in many parts of the world. The disease is spreading in new places due to human movement into the dengue disease supporting areas. Temperature is the major climatic factor which affects the biological processes of the mosquitoes and their interaction with the viruses. In the present work, we propose a multipatch model to assess the impact of temperature and human movement in the transmission dynamics of dengue disease. The work consists of system of ordinary differential equations that describe the transmission dynamics of dengue disease between humans and mosquitoes. Human population is divided into four classes: susceptible, exposed, infectious, and recovered. Mosquito population is divided into three classes: susceptible, exposed, and infectious. Basic reproduction number *ℛ*_0_ of the model is obtained using Next-Generation Matrix method. The qualitative analysis of the model is made in terms of the basic reproduction number. Parameters used in the model are considered temperature dependent. Dynamics of vector and host populations are investigated with different human movement rates and different temperature levels. Numerical results show that proper management of human movement between patches helps reducing the burden of dengue disease. It is also seen that the temperature affects the transmission dynamics of the disease significantly.

## 1. Introduction

Dengue disease is a vector-borne viral infection that usually occurs in tropical and subtropical countries. Nowadays, the disease has been recognized in over 100 countries and an estimated 50–100 million dengue cases occur annually. The disease is threatening about 40% of the world's population [1]. The disease is transmitted by the bites of infected mosquitoes named* Aedes aegypti* and* Aedes albopictus*. Four serologically different viruses* DEN* 1–*DEN* 4 cause the disease. Infection from one serotype grants life-long immunity to that strain and also shows temporary cross-immunity to the others. However, ultimately the recovered patient will become more susceptible to the other three forms [2, 3]. We assume that the infectivity of the mosquitoes ends with their death since they have a short lifespan.

Mathematical modeling has become an interesting tool for the understanding of epidemic diseases and to propose strategies to control the transmission of the disease. In 1927, Kermack and McKendrick developed an SIR model to describe epidemic diseases [4]. The model is being followed by many researchers to investigate the transmission dynamics of infectious diseases with some modifications. Esteva and Vargas proposed the SIR model to address dengue disease transmission considering constant and variable human populations [5, 6]. Since then many mathematical models have been proposed to study different aspects of dengue disease transmission. Authors [7, 8] discussed the role of awareness in controlling dengue disease transmission. Pinho et al. used a mathematical model for dengue disease transmission analysis comparing two dengue epidemics [9]. Authors [10–14] focused on incubation period to study dengue disease transmission. Sardar et al. discussed a mathematical model of dengue disease transmission with memory. They incorporated memory in the model by using a fractional differential operator [15].

Dengue infections are sensitive to the climate. Changing climate factors affect the potential for the geographic spread and future dengue disease. One of the principal determinants of* Aedes* mosquitoes' survival is temperature which has been associated with seasonal changes. The temperature plays an important role in the life cycle and behavior of the mosquitoes. So, mathematical studies have been made to understand the role of temperature in transmission dynamics of dengue disease. Brady et al. modeled* Aedes aegypti* and* Aedes albopictus* survival at different temperature levels in laboratory and field settings [16]. Liu-Helmersson et al. studied the vectorial capacity of* Aedes aegypti* and made investigations on the effects of temperature and implications for global dengue epidemic potential [17]. Polwiang discussed the seasonal basic reproduction number of dengue and impacts of climate on transmission of the disease [18].

Travel and transport contribute to the spread of infectious diseases like dengue in new places. So, one of the major factors contributing to the reemergence of infectious diseases is human movement from one place to the other. They help the disease in expanding their geographic range. Many mathematical models are proposed to address the impact of movement of humans and dispersal of vectors in the transmission dynamics of infectious diseases. Wang and Zhao discussed an epidemic model in patchy environment to describe the dynamics of disease spread among patches due to population dispersal [19]. An epidemic model was proposed by Wang and Mulone to describe the dynamics of disease spread between two patches due to population dispersal. They proved that reproduction number is a threshold of the uniform persistence and disappearance of the disease [20]. Arino and van den Driessche gave some analytical results for a model that describes the propagation of a disease in a population of individuals who travel between *n* patches [21]. Hsieh et al. proposed a multipatch epidemic model to study the impact of travel of humans on the spread of disease between patches with different level of disease prevalence [22]. Cosner et al. investigated the effects of human movement on the persistence of vector-borne diseases [23]. Dynamics of malaria disease was studied in patchy environment by Auger et al. They generalized Ross-Macdonald model to *n*-patches to describe the transmission dynamics of the disease [24]. Lee and Castillo-Chavez [25] and Phaijoo and Gurung [26] discussed dengue disease transmission dynamics in patchy environment.

Temperature influences dengue disease dynamics by affecting dynamics of mosquitoes and vector host interactions. Dengue disease has been spreading rapidly to new areas via human movement. So, in the present work, we propose a multipatch SEIR-SEI model of dengue disease considering the temperature dependent model parameters to study the impact of temperature and movement of humans on the persistence of dengue disease. We have considered different temperature levels and different movement rates in different patches. Basic reproduction number of the individual patches and a combined basic reproduction number are computed. Local stability of disease-free equilibrium point is proved by basic reproduction number.

## 2. Model Description and Formulation

The total human (host) population in each patch is subdivided into the classes: susceptible *S*_*i*_^*h*^, exposed *E*_*i*_^*h*^, infectious *I*_*i*_^*h*^, and recovered *R*_*i*_^*h*^. Mosquito (vector) population is subdivided into the classes: susceptible *S*_*i*_^*v*^, exposed *E*_*i*_^*v*^, and infectious *I*_*i*_^*v*^, *i* = 1,2, 3,…, *n*. Recovered class in the mosquito population is not considered due to their short lifespan.

The recruitment rate of host population is *A*_*i*_^*h*^. Susceptible hosts get infected by infectious vectors at the rate *b*_*i*_*β*_*i*_^*h*^*I*_*i*_^*v*^/*N*_*i*_^*h*^, where *b*_*i*_ is the biting rate and *β*_*i*_^*h*^ is the transmission probability from vector to host. The exposed host becomes infectious at the rate *ν*_*i*_^*h*^ after developing the symptoms. Infectious host recovers at the rate *γ*_*i*_^*h*^. Host dies naturally with the rate *d*_*i*_^*h*^. In case of vector population, susceptible vector gets infected by interaction with infectious hosts at the rate *b*_*i*_*β*_*i*_^*v*^*I*_*i*_^*h*^/*N*_*i*_^*h*^. The exposed vector becomes infectious at the rate *ν*_*i*_^*v*^ developing the symptoms of the disease. *d*_*i*_^*v*^ is the natural death rate of vectors.

Here, the model parameters *b*_*i*_, *β*_*i*_^*h*^, *β*_*i*_^*v*^, *d*_*i*_^*v*^, and *ν*_*i*_^*v*^ are temperature dependent. The temperature dependency relations are discussed below [17, 18]:

(1) biT=0.0043T+0.094321°C≤T≤32°C,βivT=0.0729T−0.903712.4°C≤T≤26.1°C,1,26.1°C<T<32.5°C,βihT=0.001044TT−12.28632.461−T,12.286°C<T<32.461°C,νivT=4+e5.15−0.123T,12°C<T<36°C,divT=0.8692−0.159T+0.01116T2−3.408×10−4T3+3.809×10−6T4,10.54°C≤T≤33.41°C.

We consider human movement between the patches. Human of patch *i* moves to patch *j* at the rate *m*_*ji*_^*C*^ and the human of patch *j* moves to patch *i* at the rate *m*_*ij*_^*C*^. Here *i*, *j* = 1,2, 3,…, *n* and *C* represents *S*, *E*, *I*, and *R*, respectively for susceptible, exposed, infectious, and recovered human movement rates.

The system of ordinary differential equations describing the present multipatch model [22] is given by

(2) dSihdt=Aih−biβihNihSihIiv+∑j=1nmijSSjh−∑j=1nmjiSSih−dihSih,dEihdt=biβihNihSihIiv+∑j=1nmijEEjh−∑j=1nmjiEEih−νih+dihEih,dIihdt=νihEih+∑j=1nmijIIjh−∑j=1nmjiIIih−γih+dihIih,dRihdt=γihIih+∑j=1nmijRRjh−∑j=1nmjiRRih−dihRih,dSivdt=Aiv−biβivNihSivIih−divSiv,dEivdt=biβivNihSivIih−νiv+divEiv,dIivdt=νivEiv−divIiv,i,j=1,2,3,…,n,  i≠j,

where

(3) $$\begin{array}{c} S_{i}^{h}\left(\;t\;\right)+E_{i}^{h}+I_{i}^{h}\left(\;t\;\right)+R_{i}^{h}\left(\;t\;\right)=N_{i}^{h}\left(\;t\;\right),\\ \left(\;Total\;\;host\;\;population\;\;of\;\;patch\;\;i\;\;in\;\;time\;\;t\;\right),\\ S_{i}^{v}\left(\;t\;\right)+E_{i}^{v}+I_{i}^{v}\left(\;t\;\right)=N_{i}^{v}\left(\;t\;\right),\\ \left(\;Total\;\;vector\;\;population\;\;of\;\;patch\;\;i\;\;in\;\;time\;\;t\;\right). \end{array}$$

## 3. Equilibrium Point and Stability Analysis

In this section, we find disease-free equilibrium (DFE) of the system of (2) and discuss its stability. An equilibrium is said to be disease-free if there is no infective population in both host and vector populations.

**Theorem 1 .**

Model (2) has a unique disease-free equilibrium.

**Proof**

In disease-free situation, *S*_*i*_^*h*^ = *S*_*i*_^*h∗*^ > 0, *S*_*i*_^*v*^ = *S*_*i*_^*v∗*^ > 0 and other variables *E*_*i*_^*h*^ = 0, *E*_*i*_^*v*^ = 0, *I*_*i*_^*h*^ = 0, *I*_*i*_^*v*^ = 0, and *R*_*i*_^*h*^ = 0 for *i* = 1,2, 3,…, *n*.

System of (2) for host population in disease-free situation can be written as

(4) $$\begin{array}{c} XS^{h*}=A^{h}, \end{array}$$

where

(5) X=diag⁡dih+∑j=1nmjiS−MS,MS=0m12S⋯m1nSm21S0⋯m2nS⋮⋮⋱⋮mn1Smn2S⋯0,Ah=A1h,A2h,…,AnhT,Sh=S1h∗,S2h∗,…,Snh∗T.

System of (2) for vector population in disease-free situation can be written as

(6) $$\begin{array}{c} YS^{v*}=A^{v}, \end{array}$$

where

(7) Y=diag⁡div,Sv=S1v∗,S2v∗,…,Snv∗T,Av=A1v,A2v,…,AnvT.

Since the matrix *X* has all off-diagonal entries negative and each column sum is positive, *X* is nonsingular *M*-matrix. Matrix *X* is irreducible as the matrix has nonzero diagonal elements. So, *X* must have positive inverse [27]. Hence, the system of (4) has a unique solution *S*^*h∗*^ = *X*^−1^*A*^*h*^ > 0.

Again, matrix *Y* is a diagonal matrix with positive diagonal elements. So, *Y*^−1^ exists with positive diagonal elements. Hence, the system of (6) has a unique solution *S*^*v∗*^ = *Y*^−1^*A*^*v*^ and system (2) has a unique disease-free equilibrium.

*Basic Reproduction Number*. When a typical infective is introduced into a completely susceptible population, the expected number of new infections produced by this single infective during its infectious period is called basic reproduction number.

To find the mathematical expression for the basic reproduction number, we order the variables related to the infections by *E*_1_^*h*^, *E*_2_^*h*^,…, *E*_*n*_^*h*^, *E*_1_^*v*^, *E*_2_^*v*^,…, *E*_*n*_^*v*^, *I*_1_^*h*^, *I*_2_^*h*^,…, *I*_*n*_^*h*^, *I*_1_^*v*^, *I*_2_^*v*^,…, *I*_*n*_^*v*^. We use Next-Generation Matrix method [28, 29] to find transmission matrix, *F*, and transition matrix, *V*, and we find basic reproduction number *ℛ*_0_ as

(8) $$\begin{array}{c} R_{0}=\rho \left\{\;FV^{-1}\;\right\}. \end{array}$$

For the system of (2),

(9) F=000diag⁡biβihNihSih∗00diag⁡biβihNihSiv∗000000000,V=V110000V2200V310V3300V420V44.

Here,

(10) V11=∑j≠1mj1E+ν1h+d1h−m12E⋯−m1nE−m21E∑j≠2mj2E+ν2h+d2h⋯−m2nE⋮⋮⋱⋮−mn1E−mn2E⋯∑j≠nmjnE+νnh+dnh,V22=diag⁡νiv+div,V31=diag⁡−νih,V33=∑j≠1mj1I+γ1h+d1h−m12I⋯−m1nI−m21I∑j≠2mj2I+γ2h+d2h⋯−m2nI⋮⋮⋱⋮−mn1I−mn2I⋯∑j≠nmjnI+γnh+dnh,V42=diag⁡−νiv,V44=diag⁡div.

Matrices *V*_11_ and *V*_33_ are irreducible nonnegative *M*-matrices. So, *V*_11_^−1^ and *V*_33_^−1^ exist. Also, *V*_22_, *V*_31_, *V*_42_, and *V*_44_ are diagonal matrices. So, their inverses exist. Hence, *V*^−1^ exists and basic reproduction number, *ℛ*_0_, is given by

(11) $$\begin{array}{c} R_{0}=\rho \left\{\;FV^{-1}\;\right\}. \end{array}$$

**Theorem 2 (local stability).**

The disease-free equilibrium point of the system of (2) is locally asymptotically stable if *ℛ*_0_ < 1 and unstable if *ℛ*_0_ > 1.

**Proof**

Jacobian matrix for the system of (2) at disease-free equilibrium is given by

(12) $$\begin{array}{c} \zeta =\left[\begin{array}{cc} A & B\\ 0 & F-V \end{array}\right]. \end{array}$$

Matrix *ζ* is triangular matrix. So, the stability of the system of (2) depends on matrices *A* and *F* − *V*. Matrix *A* can be written as

(13) $$\begin{array}{c} A=\left[\begin{array}{cc} -X & 0\\ 0 & -Y \end{array}\right]. \end{array}$$

Matrices *X* and *Y* (defined in Theorem 1) are nonsingular *M*-matrices. So, the matrix *A* has eigenvalues with negative real parts [27]. Hence, the stability of model (2) depends on the matrix *F* − *V* only. Here, matrix *F* is nonnegative matrix and *V* is a nonsingular *M*-matrix. So, the matrix will have eigenvalues with negative real parts if *ρ*{*FV*^−1^} < 1 [29]; that is, *ℛ*_0_ < 1. Thus, the disease-free equilibrium is locally asymptotically stable if *ℛ*_0_ < 1. If *ℛ*_0_ > 1, then *s*(*F* − *V*) > 0. Which shows that at least one eigenvalue lies in right half plane. So, the disease-free equilibrium is unstable if *ℛ*_0_ > 1.

When only the two patches are taken into the consideration, the basic reproduction *ℛ*_0_ is given by

(14) $$\begin{array}{c} R_{0}=\sqrt{\frac{1}{2}\left(m_{1}R_{01}^{2}+m_{2}R_{02}^{2}\right)+\frac{1}{2}\sqrt{{\left(m_{1}R_{01}^{2}+m_{2}R_{02}^{2}\right)}^{2}-4m_{3}R_{01}^{2}R_{02}^{2}}}, \end{array}$$

where

(15) R01=b12S1h∗S1v∗β1hβ1vν1hν1vd1vN1h2d1h+m21I+γ1hd1h+m21E+ν1hd1v+ν1v,R02=b22S2h∗S2v∗β2hβ2vν2hν2vd2vN2h2d2h+m12I+γ2hd2h+m12E+ν2hd2v+ν2v,m1=g1n1m12Im21Eν2h+ν1hg2n2ν1h−m12Im21I+g1g2−m12Em21E+n1n2,m2=g2n2m12Em21Iν1h+g1n1ν2hν2h−m12Im21I+g1g2−m12Em21E+n1n2,m3=g1n1g2n2m12Iγ1h+g3d2h+g3γ2h+g2d1hm12Eν1h+n3d2h+n3ν2h+n2d1h,g1=d1h+m21I+γ1h,g2=d2h+m12I+γ2h,g3=m21I+γ1hn1=d1h+m21E+ν1h,n2=d2h+m21E+ν2h,n3=m21E+ν1h.

Here, *ℛ*_01_ is the basic reproduction number of patch 1 and *ℛ*_02_ is the basic reproduction number of patch 2.

## 4. Simulations and Discussion

Temperature plays a significant role in the transmission dynamics of dengue disease. Small change in temperature can affect whole dynamics of the disease. Human movement from one place to the other helps spreading disease into new areas and influences the prevalence of the disease. Thus, both temperature and human movement have a significant influence on the transmission dynamics of dengue disease. For the simulation purpose, the following data are used: *N*_1_^*h*^ = 50000, *d*_1_^*h*^ = *d*_2_^*h*^ = 0.00004029, *ν*_1_^*h*^ = *ν*_1_^*h*^ = 0.1667, *γ*_1_^*h*^ = *γ*_2_^*h*^ = 0.0714, *N*_2_^*h*^ = 20000. The parameters *b*_*i*_, *β*_*i*_^*h*^, *β*_*i*_^*v*^, *d*_*i*_^*v*^, *ν*_*i*_^*v*^ are considered temperature dependent following [17].

**Figure 1:**
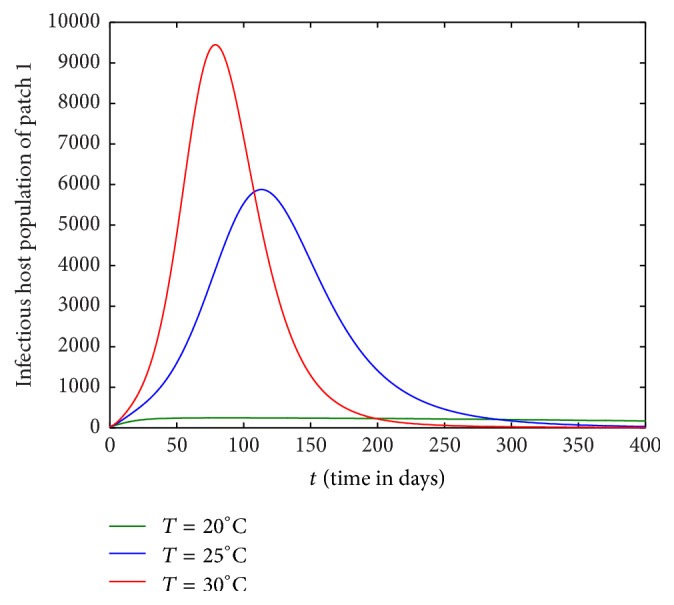
Dynamics of infectious hosts of patch 1 without host movement between the patches.

**Figure 2:**
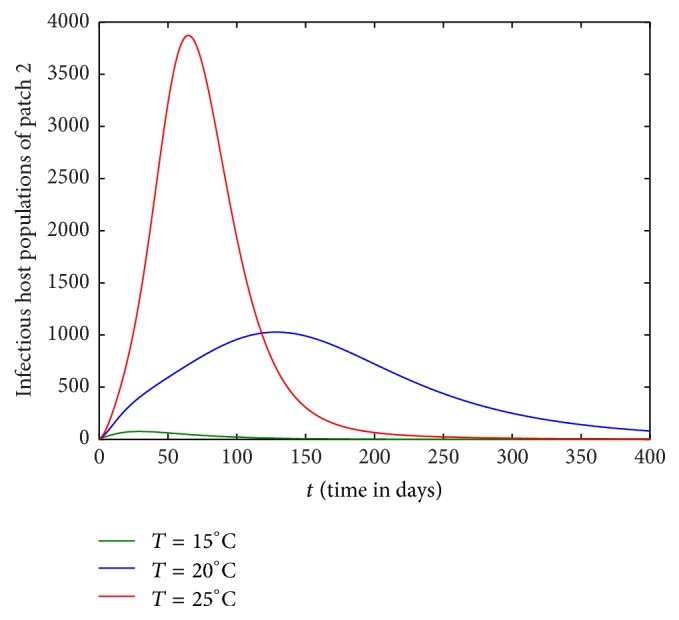
Dynamics of infectious hosts of patch 2 without host movement between the patches.

**Figure 3:**
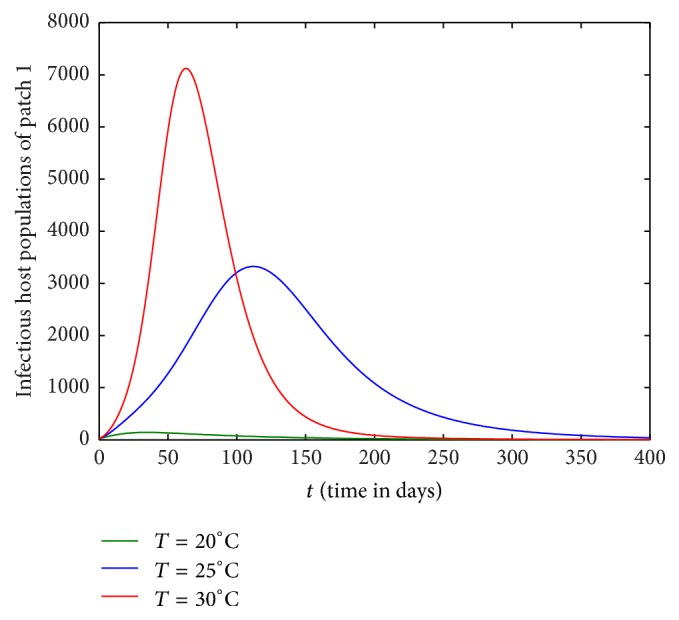
Dynamics of infectious hosts of patch 1 with host movement between patches.

**Figure 4:**
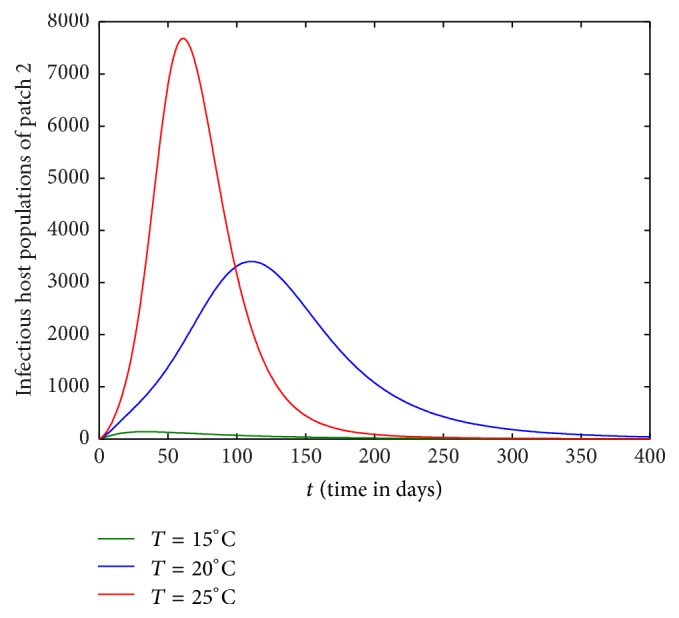
Dynamics of infectious hosts of patch 2 with host movement between patches.

Figures 1–4 are drawn with different temperature levels to investigate the dynamics of infectious hosts of patch 1 and patch 2. Figures 1 and 2 are drawn when there is no human movement between the patches. Here, patch 1 is high disease prevalent compared to patch 2. With the human movement, it is seen that infectious host population is decreased in patch 1 and the population is increased in patch 2. Thus, the human movement can cause the low endemic patch to be high endemic and high endemic patch to be low endemic patch (Figures 1–4). Also, the figures show that the burden of disease is increased with temperature. Again, the number of infectious hosts is seen increasing initially due to interaction of hosts with infectious vectors. Afterwards the number is seen decreased due to recovery from the disease and natural death of hosts (Figures 1–4).

**Figure 5:**
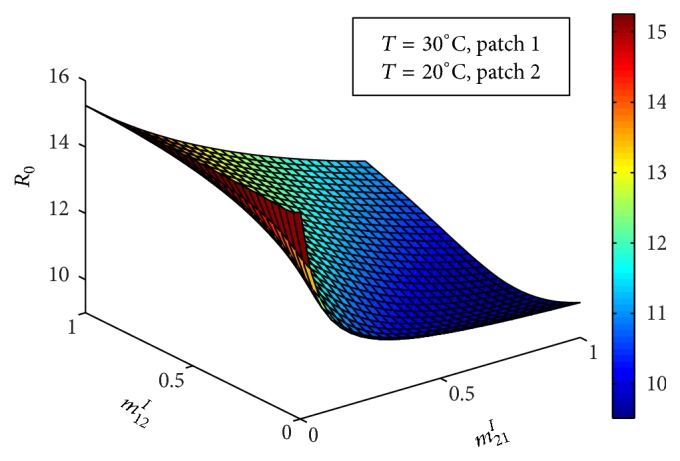
Combined basic reproduction number against *m*_21_^*I*^ and *m*_12_^*I*^.

**Figure 6:**
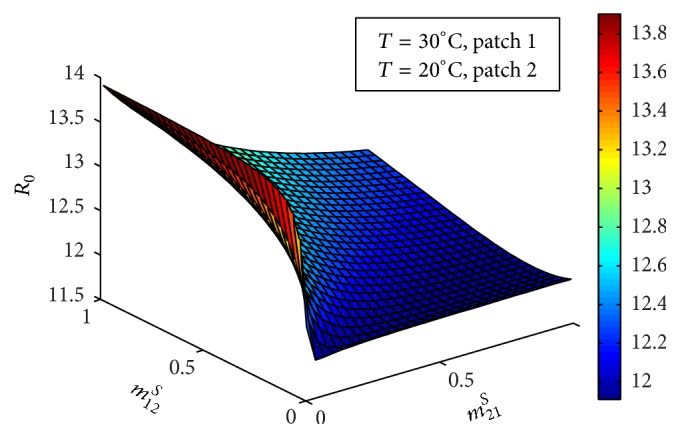
Combined basic reproduction number against *m*_21_^*S*^ and *m*_12_^*S*^.

Figures 5 and 6 show the impact of movement of infectious and susceptible hosts, respectively, on basic reproduction number *ℛ*_0_. Infectious host can infect the mosquitoes of the patch where the hosts are travelling and the susceptible hosts can get infected of the disease from the mosquitoes of the patch where the hosts have travelled. It is observed that movement of both infectious and susceptible hosts from low prevalent patch to the high prevalent patch increases the endemic level of the disease. But their movement from high prevalent patch to the low prevalent patch decreases the endemic level of the disease.

**Figure 7:**
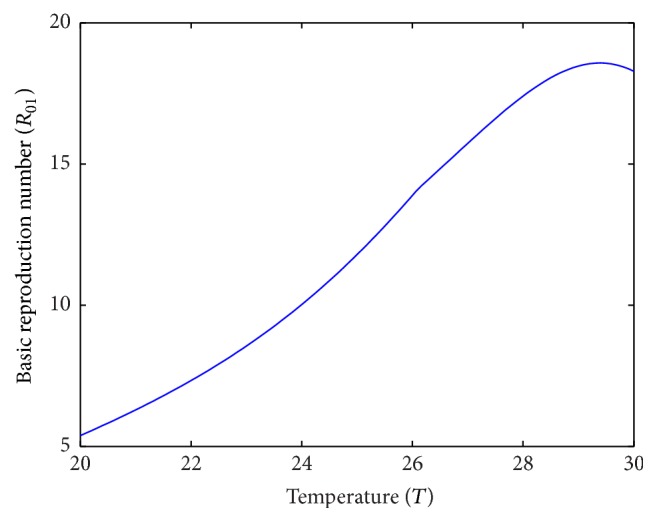
Basic reproduction number of patch 1 without host movement.

**Figure 8:**
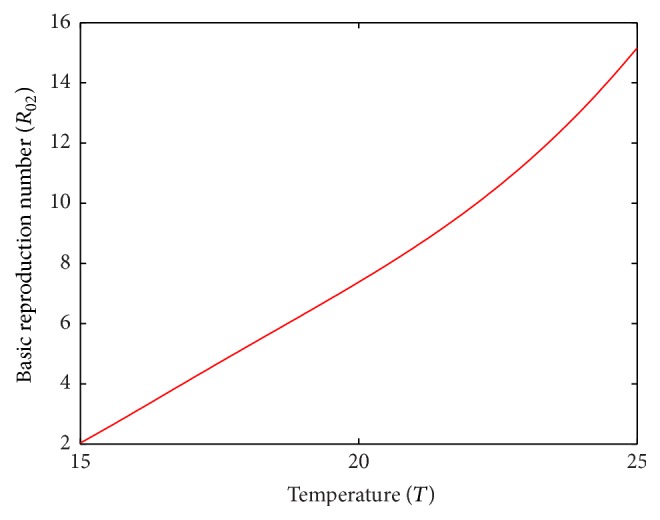
Basic reproduction number of patch 2 without host movement.

Temperature has a significant influence on basic reproduction number (Figures 7 and 8). In patch 1, the prevalence of disease is seen increasing with temperature and the maximum disease prevalence has occurred at 29.3°C temperature as in [17]. In case of patch 2 where average temperature range is 15°C to 25°C, disease prevalence increases with the increase in temperature and the maximum disease prevalence has occurred at 25°C.

### 4.1. Dynamics with Unidirectional Movement

**Figure 9:**
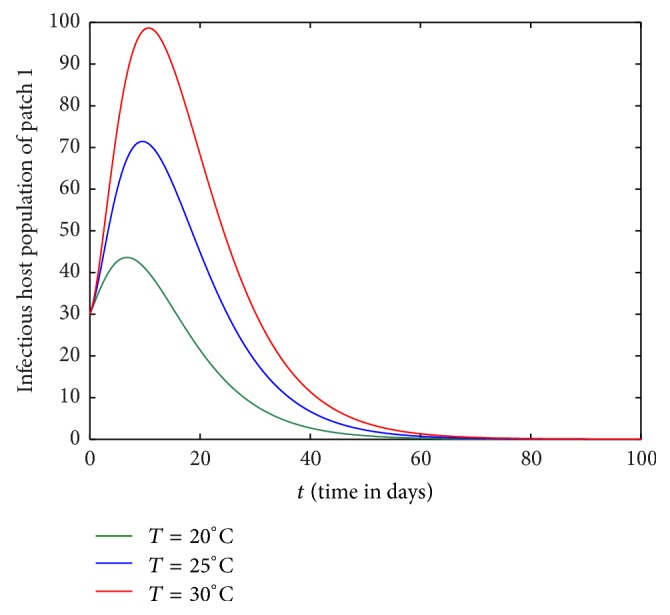
Dynamics of infectious hosts of patch 1 without host movement from patch 2 to patch 1.

**Figure 10:**
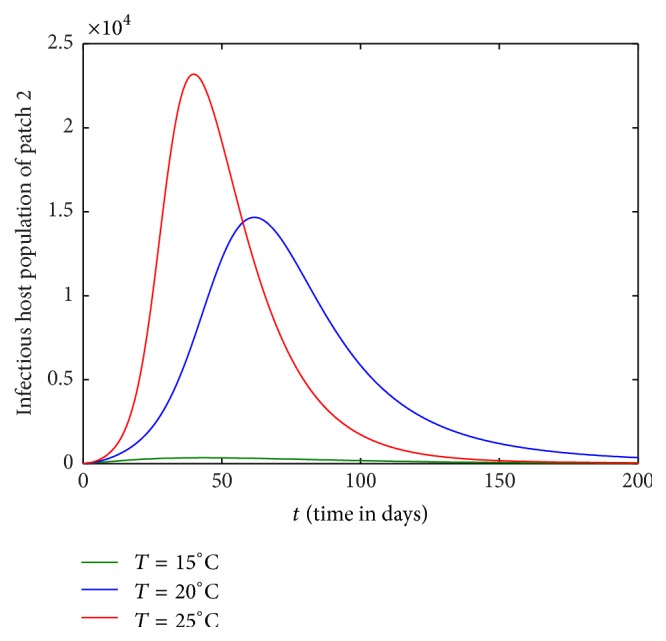
Dynamics of infectious hosts of patch 2 without host movement from patch 2 to patch 1.

**Figure 11:**
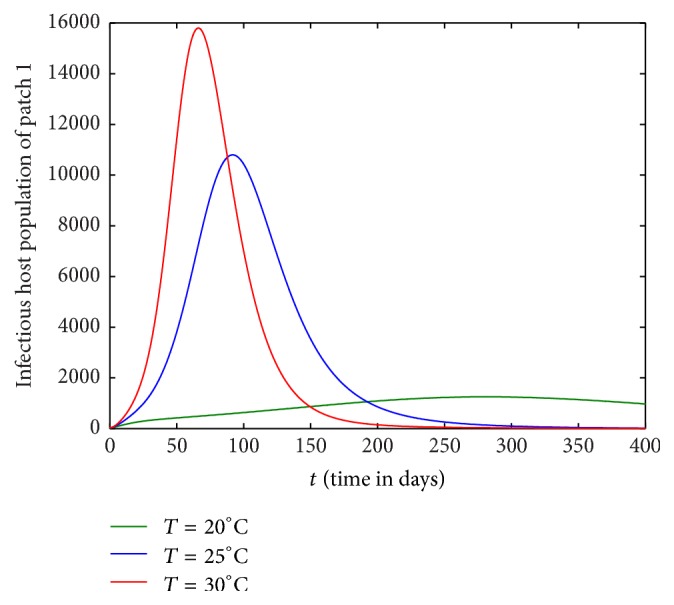
Dynamics of infectious hosts of patch 1 without host movement from patch 1 to patch 2.

**Figure 12:**
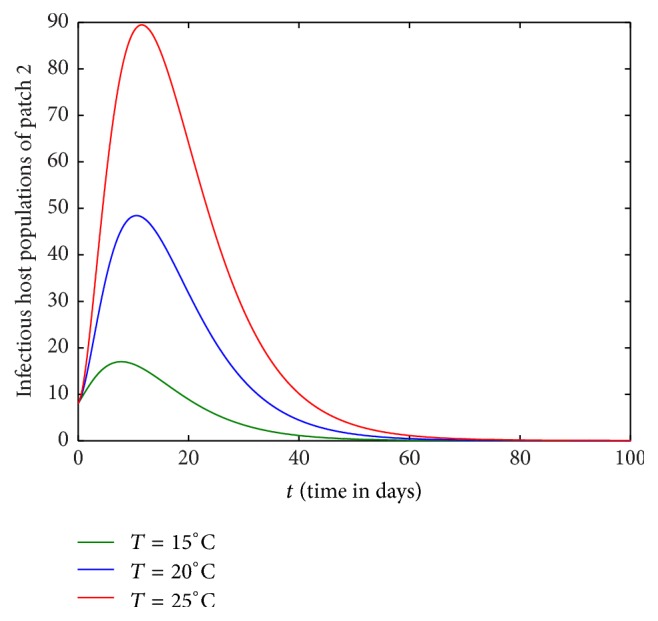
Dynamics of infectious hosts of patch 2 without host movement from patch 1 to patch 2.

In this section, we investigate the impact of host movement in one direction only with different temperature levels. Figures 9–12 show the dynamics of infectious host population of patch 1 and patch 2 when there is host movement from patch 1 to patch 2 only or patch 2 to patch 1 only. When the hosts from patch 2 are not allowed to move to patch 1 (Figures 9 and 10), burden of disease is decreased in patch 1 and increased in patch 2. When the hosts from patch 1 are restricted to travel to patch 2, the burden of disease is increased in patch 1 and decreased in patch 2 (Figures 11 and 12). In each case, the dynamics of infectious hosts are seen temperature dependent. Disease prevalence is observed increasing with temperature. Thus, movement of hosts can cause the patch to be less disease prevalent (Figures 9 and 12) and more disease prevalent (Figures 10 and 11).

**Figure 13:**
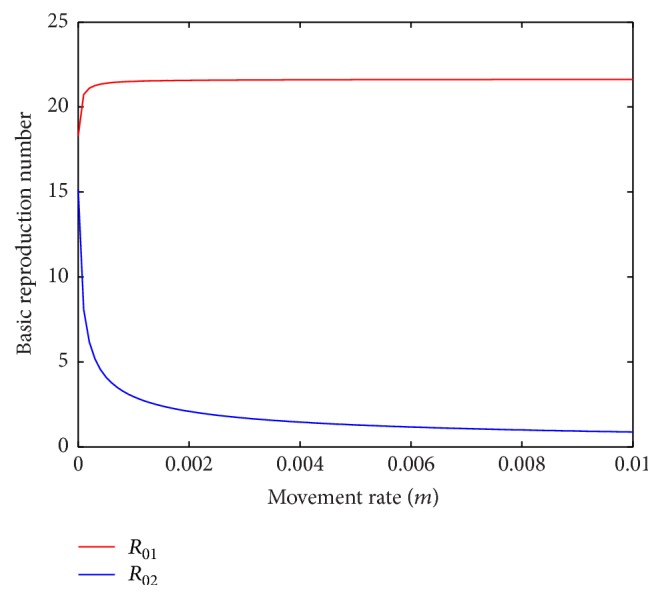
Basic reproduction numbers of patch 1 and patch 2 against movement rate, *m* = *m*_12_^*S*^ = *m*_12_^*E*^ = *m*_12_^*I*^, without host movement from patch 1 to patch 2.

**Figure 14:**
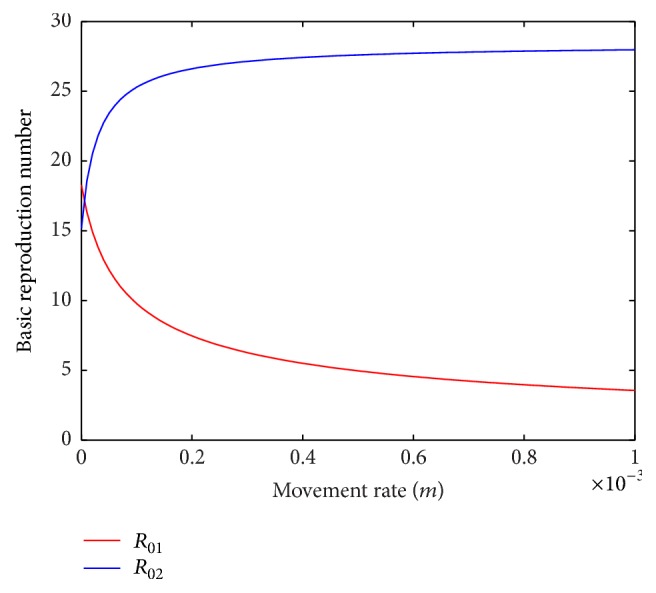
Basic reproduction numbers of patch 1 and patch 2 against movement rate, *m* = *m*_21_^*S*^ = *m*_21_^*E*^ = *m*_21_^*I*^, without host movement from patch 2 to patch 1.

When only the hosts from patch 2 are allowed to move to patch 1, basic reproduction number of patch 1 increases and that of patch 2 decreases with the increase in movement rate (Figure 13). Also, basic reproduction number of patch 1 decreases and that of patch 2 increases when only the hosts from patch 1 are allowed to move to patch 2 (Figure 14). So, the host population can be moved from one patch to the other to bring the disease under control.

## 5. Conclusion

Temperature plays a significant role in dynamics of dengue disease transmission. It affects the lifecycle and biting behavior of mosquitoes. Human movements contribute in spreading the disease in new places. We have proposed multipatch model of dengue disease with the human movement between patches considering temperature dependent model parameters. In the present work, we explored the impact of temperature and host movement between patches on the transmission dynamics of dengue disease. We have investigated the stability of disease-free equilibrium point. It is observed that the point is locally asymptotically stable when basic reproduction number *ℛ*_0_ < 1 and unstable when *ℛ*_0_ > 1. Simulated results show that basic reproduction number depends on temperature and host movement. The prevalence of disease can increase or decrease with temperature and mobility of hosts from one patch to the other. Present work shows that the burden of the disease can be reduced by managing the host movement and the temperature can enhance the strength of the disease. These pieces of information can be helpful to the concerned authorities to bring dengue disease under control.
